# Chicago

**Published:** 1899-11

**Authors:** Joseph B. Clancy

**Affiliations:** Secretary


Secretary.

### CHICAGO VETERINARY SOCIETY.

The fourth annual meeting was called to order by President Robertson on Thursday evening, October 19th. The subjects presented were so intensely interesting that the twenty members present could not resolve to adjourn until a very few minutes before midnight.

The President called the attention of the members to the meeting of the Illinois State Veterinary Medical Association, to be held in Chicago during the month of November, and he hoped that the Entertainment Committee of the Veterinary Society would provide a programme that would prove interesting, as in all probability a joint meeting of the two Societies would be arranged for, at which a clinic would be one of the main features. All who had cases of interest at the time of meeting were invited to present them. In closing his remarks he said the passing of the bicycle as a competitor of the horse he felt was an assured fact, and at present he opined that there is nothing to fear from the automobiles.

The application of Dr. Wm. C. Siegmund for membership in the Society received the favorable indorsement of the Board of Censors, and his election as a member of the Society followed.

The election of officers for the ensuing year resulted on the selection of the following by a unanimous vote: Dr. Joseph Hughes, President; Dr. L. A. Merillat, First Vice-President; Dr. A. C. Worms, Second Vice-President; Dr. H. W. Hawley, Third Vice-President; Dr. Joseph B. Clancy, Secretary; Dr. R. G. Walker, Treasurer.

Dr. Robertson, on retiring from the chair, presented the Society's new President, and recalled the efforts of the past few years to have him consent to his election to the office; his excuse had been sustained by the members; now it is overruled, and we may congratulate ourselves at his acceptance.

President Hughes rehearsed his excuse for declining in the past, it being his opinion that some one not so directly connected with either of the veterinary colleges should fill the office. He, therefore, came to the meeting unprepared for the honor bestowed.

The appointment of committees for the current year was now in order, and the following were named: Board of Censors—Drs. E. L. Quitman, Frank Allen, and O. R. Dubia. Entertainment Committee—Drs. Robertson, Worms, and E. L. Quitman. Legislative Committee—Drs. Walker, Hawley, Merillat, Ryan, and Allen. Literary and Publication Committee—Drs. A. H. Baker, A. M. Casper, L. Campbell, and the Secretary *ex-officio*.

Dr. H. W. Hawley reported a case of "Rupture of the Tendon of the Metatarsi Flexor," and announced that all who wished to see it were welcome to do so. An interesting discussion followed, in which Dr. Jos. Hughes described the method of operating for the cure of such cases, and Dr. E. L. Quitman reported a similar case, following which many other subjects were discussed, and many were willing to continue when the motion to adjourn prevailed at 11.55 P.M.

The monthly meeting was convened at the Brevoort Hotel, on Thursday evening, November 9th. President Hughes presided, and the following responded to roll-call: Drs. Campbell, Casper, Clancy, Dubia, Donovan, Howe, Hughes, Hawley, Merillat, Nelson, Pierce, Pistor, P. Quitman, E. L. Quitman, Robertson, Siegmund, Siegroesser, Walker, Worms; and Dr. Wooden, whose application had the favorable indorsement of the Board of Censors, was elected to membership.

A motion of Dr. Robertson's prevailed, instructing the President to appoint a committee of three, who shall report at the December meeting, of some place available for the permanent meeting of the Society.

Dr. E. L. Quitman, in presenting his paper, apologized for not going into his subject more thoroughly; pressure of business had prevented him from carrying out his original plan. The paper was well received.

Dr. A. H. Baker thought that it was open to a little friendly criticism, and proceeded to express his surprise at some of the statements it contained. After a lengthy and spirited discussion, in which nearly all those present took part, it was resolved that questions brought up in the paper be laid upon the table for further consideration, and the terms "serviceably sound," "wind and work," etc., in use at the sale-ring of the Union Stockyards, are still as indefinite as ever.

"Brush Firing," Dr. Robertson announced, was something new to him, and there were others who confessed their utter and dense ignorance. Dr. William C. Siegmund explained the method and its effects, and it was contended by many that the same results could be accomplished by the use of blisters, and with less bother.

JOSEPH B. CLANCY,
Secretary.

